# Atypical Presentation of a Penile Fracture With Unilateral Corpora Cavernosa and Urethral Injury

**DOI:** 10.7759/cureus.67816

**Published:** 2024-08-26

**Authors:** Kulsoom Durrani, Zachary Corey, Alexander Birk, Susan M MacDonald

**Affiliations:** 1 College of Medicine, Penn State University, Hershey, USA; 2 Medicine, Penn State Health Milton S. Hershey Medical Center, Hershey, USA; 3 Urology, Urology of Virginia, Virginia Beach, USA; 4 Urology, Penn State Health Milton S. Hershey Medical Center, Hershey, USA

**Keywords:** urology, genital trauma, penis, urethral injury, penile fracture, fracture penis

## Abstract

A penile fracture is a rare but urgent urologic emergency that requires immediate surgical correction to preserve erectile function. It results from a rupture of the tunica albuginea of one or both corpora cavernosa. Patients classically will present with a history of recent sexual activity and a sudden "snap," followed by detumescence, pain, and swelling of the phallus. The rupture of the tunica albuginea typically happens ventrolaterally along the penile shaft, as the tunica albuginea thins at this location, especially during erections. In this report, we present a case of a penile fracture with a rupture of the tunica albuginea at an unusual location in the distal phallus between the corpora cavernosa and corpora spongiosum.

## Introduction

A penile fracture is a rare but serious urologic emergency that results from a rupture of the tunica albuginea of one or both corpora cavernosa. Patients classically present with a history of recent vigorous sexual activity during which the patient experiences a sudden "snap" with pain followed by immediate detumescence and the development of penile swelling and discoloration commonly known as the "eggplant" deformity. The reported "snap" is the rupture of the tunica albuginea, which most commonly occurs ventrolaterally along the penile shaft. Similar presentations may be mimicked by conditions such as a rupture of the superficial dorsal penile vein. Urgent surgical repair is necessary to preserve penile functionality [1-3]. 

In this report, we present a case of a penile fracture with a rupture of the tunica albuginea at an unusual location in the distal phallus between the corpora cavernosa and corpora spongiosum laterally.

## Case presentation

A 43-year-old man felt a snapping sensation during intercourse that was associated with detumescence, pain, and a large volume of blood per urethra. He initially sought care at an emergency department outside our institution, and as he had no edema or ecchymosis on the exam, he was sent home with a plan for outpatient urology follow-up. The next morning, he had urethrorrhagia during a partial nocturnal erection and presented to our emergency department. He reported voiding twice since the initial incident - once with hematuria at the beginning and end of micturition and the second with no hematuria. He also only reported pain at the distal tip of the penis. On physical exam, there was no edema, erythema, or ecchymoses of the skin overlying the phallus to suggest injury. There was blood at the meatus at rest or with palpation. His urinalysis demonstrated 50+ red blood cells on microscopic analysis.

**Figure 1:**
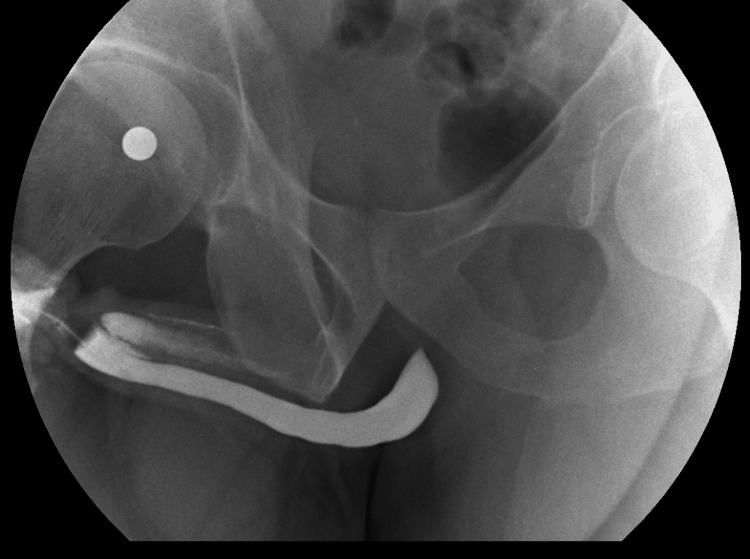
Retrograde urethrogram

**Figure 2:**
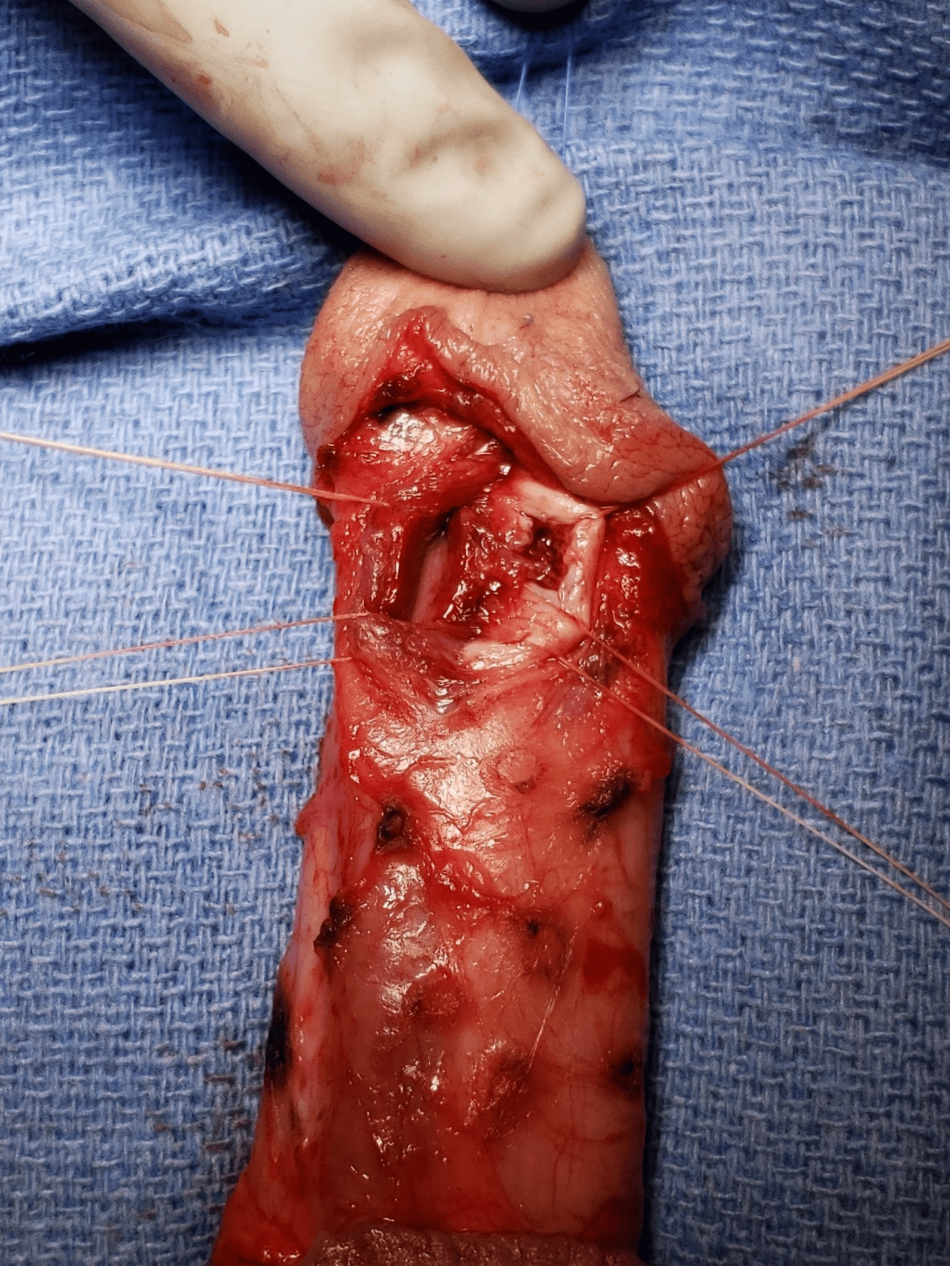
Defect

A retrograde urethrogram was performed to evaluate for urethral injury. The urethrogram demonstrated contrast extravasation into the corpora cavernosa suggesting an anomalous connection to the urethra created by his prior trauma (Figure 1). As the patient was quite young with no erectile dysfunction, he was taken for operative exploration in an effort to prevent erectile dysfunction or a fistula formation. On degloving the penis, there was no suggestion of a disruption of the tunica albuginea. During cystoscopy, a 0.5 cm linear laceration in the urethral mucosa was noted on the left dorsolateral portion of the distal urethra just above the coronal sulcus. The corpora spongiosum was dissected laterally off the left corpora cavernosa, and the glans cap was dissected 4 mm superiorly to reveal the laceration in the left corpora cavernosa and the adjoining corpora spongiosum (Figure 2). 

These injuries were successfully repaired using a two-layer running 5-0 PDS on the urethra and corpus spongiosum and interrupted 2-0 Vicryl on the corpus cavernosum. Resorbable sutures were utilized to avoid additional inflammation and scarring caused by leaving a permanent foreign body in the injury. A 16 French catheter was left indwelling for two weeks. Repeat pericatheter retrograde urethrogram confirmed no extravasation prior to catheter removal.

On the eight-month follow-up, the patient reported successfully sustaining an erection and engaging in intercourse with minimal pain. On assessment, he had no abnormal curvature of the penis and was managing minor pain during intercourse with nonsteroidal anti-inflammatory drugs (NSAIDs). He did well for a two-year interval and then subsequently developed chronic pelvic pain. Although he has no erectile dysfunction, he reports pain that is centered at the glans and distal urethra but is accompanied by perineal discomfort and muscle spasms. He has tried muscle relaxants and is working with physical therapy to improve his pelvic floor muscle dysfunction. Written informed consent was given by the patient to share his case.

## Discussion

A penile fracture most commonly occurs due to blunt trauma sustained during sexual intercourse leading to the rupture of the tunica albuginea surrounding the corpora cavernosa. Clinical diagnosis is made based on characteristic history and exam findings. Most patients report a snap or pop sound followed by immediate detumescence and pain. Physical exam typically demonstrates penile swelling and discoloration, also known as the "eggplant" deformity. These findings should prompt immediate suspicion of penile fracture and American Urological Association (AUA) guidelines recommend urgent surgical intervention to prevent adverse outcomes [4].

The annual incidence of penile fracture in the USA is estimated to be about 500 to 600 cases per year, and it accounts for one in 175,000 emergency admissions [5,6]. The rate of penile fracture associated with urethral injury varies widely depending on the geographical area and etiology of the injury. Generally, in Western countries like the USA, rates are about 21% [6], but they have been reported to be as high as 38% [7]. Findings of hematuria, blood at the urethral meatus, and voiding difficulty have been highlighted as key indicators for urethral injury and should prompt further investigation by providers based on current AUA guidelines [4]. While indicated, many patients with penile fractures and associated urethral injury are not appropriately assessed as indicated by Prasier et al. in their study of 3,883 patients. While about 21% of their patients were found to have penile fractures with urethral injury, 66% of 21% of patients with urethral injury did not undergo formal urethral evaluation [6].

The tunica albuginea surrounds both corpora cavernosa and is about 2 mm in thickness [8]. However, it thins to about 0.25 mm during erections, and the thinnest point within the tunica albuginea is ventrolaterally [8,9]. Consequently, the chance of a rupture in the tunica albuginea is highest during intercourse, as there is an increased chance of sudden flexion-based trauma at the thinnest points in the tunica. Unsurprisingly, numerous studies have corroborated that the most common etiology of penile fracture is sexual intercourse or masturbation [10-12]. Interestingly, in our case, the patient was found to have a rupture in the left tunica albuginea ventromedially, between the corpora and spongiosum. Of note, the patient denied insertion of any foreign bodies into his urethra, or urethral sounding as part of intercourse. It is likely that there were limited physical exam findings because the location of the defect prevented the creation of a visible hematoma.

Imaging modalities to help diagnose penile fracture include ultrasonography, computed tomography (CT), magnetic resonance imaging (MRI), and retrograde urethrography or urethroscopy in cases of potential urethral injury. While imaging is often helpful in establishing penile fractures, diagnosis is based on clinical findings and the patient’s history [4,13]. MRI is often costly and time-consuming [14,15], ultrasound is operator-dependent [13,15], and CT scan requires radiation and is not an ideal modality for tracing the continuity of the tunica albuginea [13]. Retrograde urethrography is an effective next step in the workup of patients with suspected urethral involvement [16]. Cystoscopy can also be utilized to identify defects in the corpora cavernosa. Both cystoscopy and retrograde urethrogram can be completed in the emergency department at the bedside prior to repair or in the operating room during surgical repair.

Clinical suspicion for penile fractures should remain high, especially if the patient endorses a textbook history, to avoid missing unusual presentations. Urgent surgical repair is the preferred treatment modality as delays in treatment can increase the rates of complications. One meta-analysis comparing immediate surgical repair versus conservative management of penile fractures found that the rate of complications in the surgical group was 46.4%, as compared to 89% in the medical management group. The study further investigated the rates of erectile dysfunction, penile curvature and development of plaques/nodules, and painful erections between patients managed surgically versus conservatively and found that the rates were significantly lower in the patients managed with surgery [11,17].

In this case, the patient presented without the classic penile fracture physical exam findings, but did have a history that was suggestive. When he was evaluated for the presence of blood at his urethral meatus, the penile fracture was diagnosed. After additional workup and treatment, he was found to have a tear in the tunica albuginea between the corpora and the spongiosum, in the ventromedial direction. This case demonstrates the need to maintain a high suspicion of penile fracture in the case of a history consistent with the diagnosis despite the lack of physical exam findings.

There are three interesting facets to this case: 1) the location of the injury, 2) the questionable mechanism of injury, and 3) the question of repair. As previously stated, fractures typically occur ventrolaterally and are visible with degloving of the phallus, but this defect in the tunica albuginea occurred medially between the cavernosum and the spongiosum. Further adding interest, the injury is very distal within the phallus. As to the mechanism, the patient was questioned about urethral sounding or the insertion of any foreign bodies, which he denied. To the third point, we considered whether to leave a catheter to allow this injury to heal as would be done in a urethral perforation during a penile implant procedure; however, as the patient was very young with excellent function, the shared decision was to intervene with the thought process being that it would be a more certain way to help prevent erectile disfunction or fistula formation.

## Conclusions

In the case presented, the tunica albuginea injury occurred medially between the cavernosum and the spongiosum rather than in the typical ventrolateral direction. Consequently, the patient lacked the classic penile fracture physical exam findings, despite endorsing a history suggestive of penile fracture. A key teaching point from this case is the need to maintain high clinical suspicion for penile fractures, especially in cases when the patient endorses a textbook history, to avoid missing unusual presentations. Urgent surgical repair is the preferred treatment modality as delays in treatment can increase the rates of complications.
